# GILT restricts the cellular entry mediated by the envelope glycoproteins of SARS-CoV, Ebola virus and Lassa fever virus

**DOI:** 10.1080/22221751.2019.1677446

**Published:** 2019-10-21

**Authors:** Danying Chen, Zhifei Hou, Dong Jiang, Mei Zheng, Guoli Li, Yue Zhang, Rui Li, Hanxin Lin, Jinhong Chang, Hui Zeng, Ju-Tao Guo, Xuesen Zhao

**Affiliations:** a Institute of Infectious disease, Beijing Ditan Hospital, Capital Medical University, Beijing, People’s Republic of China; b Beijing Key Laboratory of Emerging Infectious Disease, Beijing, People’s Republic of China; c Department of Pulmonary and Critical Care Medicine, General Hospital of Datong Coal Mine Group Co., Ltd., People’s Republic of China; d Department of Pathology and Laboratory Medicine, Western University, London, Ontario, Canada; e Baruch S. Blumberg Institute, Hepatitis B Foundation, Doylestown, PA, USA

**Keywords:** Interferon-stimulated genes (ISGs), GILT, SARS-CoV, Ebola virus, Lassa fever virus

## Abstract

Interferons (IFNs) control viral infections by inducing expression of IFN-stimulated genes (ISGs) that restrict distinct steps of viral replication. We report herein that gamma-interferon-inducible lysosomal thiol reductase (GILT), a lysosome-associated ISG, restricts the infectious entry of selected enveloped RNA viruses. Specifically, we demonstrated that GILT was constitutively expressed in lung epithelial cells and fibroblasts and its expression could be further induced by type II interferon. While overexpression of GILT inhibited the entry mediated by envelope glycoproteins of SARS coronavirus (SARS-CoV), Ebola virus (EBOV) and Lassa fever virus (LASV), depletion of GILT enhanced the entry mediated by these viral envelope glycoproteins. Furthermore, mutations that impaired the thiol reductase activity or disrupted the N-linked glycosylation, a posttranslational modification essential for its lysosomal localization, largely compromised GILT restriction of viral entry. We also found that the induction of GILT expression reduced the level and activity of cathepsin L, which is required for the entry of these RNA viruses in lysosomes. Our data indicate that GILT is a novel antiviral ISG that specifically inhibits the entry of selected enveloped RNA viruses in lysosomes *via* disruption of cathepsin L metabolism and function and may play a role in immune control and pathogenesis of these viruses.

## Introduction

In recent decades, cross-species transmission of numerous zoonotic viruses causes severe diseases in infected individuals and poses formidable threats to public health globally [1–3]. For example, outbreak of severe acute respiratory syndrome (SARS) in 2002/2003, caused by SARS coronavirus (SARS-CoV) transmitted from wildlife animal reservoirs, was associated with 774 deaths in 8096 infected individuals [4]. Ebola virus (EBOV) and Lassa virus (LASV) that cause Ebola disease and Lassa fever diseases, which are characterized with severe haemorrhagic fever, multi-organ failure, and high mortality rate, are currently circulating in West Africa and remain to be big challenges to medical community [5,6].

The interferon (IFN)-mediated innate immune response provides a robust first line of defence against virus infection through the induction of hundreds of interferon-stimulated genes (ISGs) that establish an antiviral state in host cells [7]. Many ISGs are cell-intrinsic restriction factors that are constitutively expressed in barrier cells such as epithelial cells, endothelial cells and macrophages and restrain viral infection by directly targeting various stages of virus life cycle [7]. So far, several ISGs, including interferon-induced transmembrane proteins (IFITM), cholesterol-25-hydroxylase (CH25H), ArfGAP with dual pleckstrin homology domains 2 (ADAP2) and a short isoform of nuclear receptor coactivator 7 (NCOA7) have been identified to block the viral entry by inhibiting virus-host cell membrane fusion or endocytic trafficking of internalized virions [8–13]. Some other ISGs target post-entry steps of viral life cycle, as exemplified by TRIM5α and Mx that block the nucleocapsid uncoating, RNase L and ISG20 that degrade viral RNA, PKR that inhibits viral protein translation, BST2 and Viperin that inhibit virion assembly or budding (reviewed in [7]). Although the critical role of these antiviral ISGs in control of viral infections has been demonstrated in animal models and human diseases [14], the roles and mechanisms of many other ISGs in controlling virus infections remain to be determined.

Gamma-interferon-inducible lysosomal thiol reductase (GILT, also named IFI30) is a soluble thiol reductase that is abundantly expressed in the lysosome of professional antigen-presenting cells (APCs), such as macrophages, dendritic cells and B lymphocytes, and can be induced by IFN-γ in other cell types [15,16]. Upon translation, GILT is sorted by mannose 6-phosphate receptor pathway to endocytic compartments and finally transported into the lysosome that provides optimal low pH for its thiol reductase activity [16,17]. As the only thiol reductase in endolysosomes and phagosomes, GILT contributes to the degradation of lysosomal protease cathepsin, maintenance of cellular normal redox state and prohibition of aberrant autophagy [18–21]. GILT utilizes two cysteine residues in the CXXC reductase motif to reduce the disulphide bond of endocytosed antigens to facilitate antigen processing and presentation by MHC II and MHC-I [16,17,22–24]. Hence, GILT has been demonstrated to play an essential role in the cross-priming of viral-specific T-cell response [22]. However, its role in intrinsic and innate antiviral immunity has not been explored.

Besides directly fusing with cytoplasm membrane, enveloped viruses enter cells by fusion with cell membranes in different endocytic compartments. While Vesicular stomatitis virus and HCV enter host cells via Rab5^+^ early endosome, many flavivirus and influenza A virus invade cells through Rab7^+^ late endosome (reviewed in [25]). Notably, SARS-CoV and EBOV are transported to lysosomes to fulfil infectious entry [26]. Given that GILT plays a crucial role in unfolding internalized viral glycoproteins and affecting the environment in the lysosome [22,27], an cellular compartment utilized by SARS-CoV, EBOV and many other viruses to invade into the cytoplasm of host cells and initiate viral replication [26,28–30], it is conceivable that GILT may affect the infectious entry of this group of viruses. In this study, we demonstrated that GILT is constitutively expressed in lung epithelial cells and restricts the entry mediated by envelope glycoproteins (GP) from SARS-CoV, EBOV and LASV. We also found that mutations altering two cysteine residues in CXXC motif or abrogating N-linked glycosylation impaired GILT-mediated restriction, implying that lysosomal localization and thiol reductase function are essential for GILT to inhibit the entry of these viruses. Our study thus revealed an important role of GILT in the control and pathogenesis of selected RNA virus infections.

## Materials and methods

### Cell lines, IFNs and antibodies

293T and A549 cells were cultured with Dulbecco’s modified Eagle’s medium (DMEM; Invitrogen). MRC-5 cells were cultured with Eagle's Minimum Essential Medium (MEM; Corning). THP-1 cell line was grown in RPMI-1640 medium (Invitrogen). All media were supplemented with 10% fetal bovine serum (FBS), penicillin G, streptomycin and 2 mM L-glutamine (Invitrogen). FLP-IN T Rex 293 cells were maintained in complete DMEM supplemented with 10 μg/ml blasticidin (Invitrogen) and 100 µg/ml Zeocin (Invitrogen).

Human Interferon alpha-2b (IFN-α2b) and IFN-γ were ordered from PBL Interferon Source (Catalog No. 11105–1 and 11500-1, respectively). Monoclonal antibody against FLAG tag (ANTI-FLAG M2) and β-Actin were purchased from Sigma (Catalog No. F1804 and A2228, respectively). Rabbit polyclonal antibody against GILT was obtained from Sigma (HPA026650). GILT Monoclonal antibody (G-11) was obtained from Santa Cruz (sc-393507A). Rabbit polyclonal antibody targeting cathepsin L (10938-1-AP) and B (12216-1-AP) were ordered from Proteintech. Anti-cathepsin S (ab134157) was obtained from Abcam.

### Plasmid construction

GILT (NM_006332) cDNA Clone was purchased from Origene (RC205877) and cloned into pcDNA5/FRT/ΔCAT vector between BamH I and Not I sites. Plasmids expressing mutant GILT protein with point mutations were constructed by overlap extension PCR [12,31]. By using the template of pcDNA3/ACE2-C9 reported previously [32], human ACE2 with C-terminally C9 tag was subcloned into retroviral vector pCX_4_bsr vector between BamH I and Not I sites. LentiCRISPRv2/gilt plasmids were constructed using methods previously described [33]. Briefly, Guide RNA encoding oligonucleotides were annealed and cloned into BsmBI-linearized plentiCRISPRv2. The oligonucleotides used were listed in following (forward/reverse): for sgRNA1, caccgCCTACCTTGTAGTTAACTGG/aaacCCAGTTAACTACAAGGTAGGc; for sgRNA2, caccgACTTCTTCAGGGGCCCCCGC/aaacGCGGGGGCCCCTGAAGAAGTc.

### Pseudotyped retroviral particles for transduction and infections

Plasmids and procedures used to generate the variety of viral envelope protein-pseudotyped lentiviruses bearing luciferase reporter genes were previously reported [32,34]. Also, VSV G protein-pseudotyped retroviruses expressing wild-type GILT or human ACE2 were packaged in GP2-293 cells with previously described methods [35].

To package VSV-G-pseudotyped HIV-1 lentiviral particles for knockout of *GILT* in THP-1 cells, Lenti-X 293T cells grown at 90% confluence in 100-mm-diameter dish were cotransfected with 12 µg of plentiCRISPRv2 plasmids targeting GILT (LentiCRISPRv2/gilt) or empty vector (LentiCRISPRv2/CTRL), 9 µg of psPAX2 (Addgene) and 3 µg of pCMV/VSV-G using Lipofectamine 2000. The virus was harvested at 48 and 72 h after transfection, filtered through PES filters, and pooled together. The collection was concentrated using Lenti-Pac^TM^ lentivirus concentration solution (GeneCopoeia^TM^) and stored at −80°C until use.

### BlaM-Vpr based viral entry assay

The Blam-Vpr based entry assay was applied to study the effect of IFN-γ on viral entry as described previously [36]. Briefly, 293T cells were cotransfected with pNL4-3. Luc. R^-^ E^-^, pCMV-Blam-Vpr (NIH AIDS Research and Reference Reagent Program), pAdVAntage vector (Promega), and plasmids encoding viral GP protein to produce Blam-Vpr chimera pseudoviral particles. A549 cells were treated with IFN-γ and inoculated with Blam-Vpr pseudoviral particles. CCF2 substrate was loaded into cells at 1 h after infection and then washed three times with PBS. At 24-h postinfection, the cells were fixed with 2% formaldehyde and analysed by flow cytometry.

### Establishment of cell lines stably expressing ACE2 and GILT protein

As previously described [35], A549-derived cell lines stably expressing ACE2 (A549/ACE2) were established by spin-inoculation of ACE2 pseudotyped retroviruses and blasticidin (6 µg/ml) selection. The resulting A549/ACE2 cell line was subsequently transduced with GILT pseudotyped retroviruses and selected with 2 µg/ml puromycin for 2 weeks. The puromycin and blasticidin dual resistant cells were expanded to generate cell lines stably expressing ACE2 and GILT proteins. FLP-IN T Rex 293-derived cell lines expressing wild-type or mutant GILT proteins in a tetracycline (Tet)-inducible manner were established as previously described [32,35].

### Generation of CRISPR/Cas9 THP-1 cell clones

Similar to previous report [33], THP-1 cells were spin-infected with concentrated LentiCRISPRv2/*GILT* pseudovirus in the presence of 40 μg/ml DEAE-Dextran. Forty-eight hours after transduction, the transduced THP-1 cells were selected with 1 μg/ml of puromycin for two weeks. Single-cell clones were generated by limiting dilution and puromycin selection. The resulting clones were screened with western blot and validated by genomic DNA sequencing. Two pairs of (forward/reverse) primers 5′-GATGACCCTGTCGCCACTTC-3′/5′-CAGTAGGCGCTCATTGAACC-3′ and 5′-TGAACCAGGGAGTCGGGTGT-3′/5′-GCAAGGCAGCAGGGTGAGAG-3′ were used to amplify gRNA-targeted exons 1 and 2, respectively. The amplicons cloned into pGEM-T vectors was sequenced and analysed using Clustal W program.

### Western blot assay

Cell monolayers were rinsed with 1× phosphate buffered saline (PBS) and lysed with 1× Laemmli buffer. An aliquot of cell lysate was separated on NuPAGE® Novex 4-12% Bis-Tris Gel (Invitrogen) and transferred onto a PVDF membrane. The membranes were blocked with PBS containing 5% nonfat dry milk and the expression of GILT or cathepsins was probed with the GILT polyclonal antibody (HPA026650) or cathepsin antibody at 1:1000 dilution. The bound antibodies were visualized with IRDye secondary antibodies (1:10,000) and imaging with LI-COR Odyssey system.

### Luciferase assay

FLP-IN T REX 293**-**derived GILT-expressing cell lines were transfected with plasmids encoding ACE2, APN or DPP4 to express viral receptor, and seeded into 96-well plates with black wall and clear bottom. THP-1-derived cell lines, 0.8 × 10^5^ cells per well were seeded into black wall 96-well plates and treated with PMA (10 ng/ml) for 24 h to induce differentiation. The differentiated cells were infected with desired pseudotyped lentiviral particles for 4 h, and then replenished with fresh media. Two days post infection, the media were removed, and cells were lysed with 30 µl/well of cell lysis buffer (Promega) for 15 min, followed by adding 50 µl/well of firefly luciferase substrate (Promega). The firefly luciferase activities were determined by luminometry in a TopCounter (Perkin Elmer).

### Immunofluorescence

To visualize the subcellular localization of wild-type and mutant GILT proteins, A549 or TRex 293 cells were first fixed with 4% paraformaldehyde for 20 min and then permeabilized with 0.1% Triton X-100 for 10 min. After incubation in the blocking solution (1×PBS with 3% BSA and 5% FBS) for 1 h, cells were stained with a mouse monoclonal antibody (G-11, SANTA CRUZ sc-393507A) or rabbit polyclonal antibody recognizing GILT protein (sc-393507A) together with a Rabbit derived monoclonal antibody against EEA1 (Cell Signaling, 2411), Rab9 (Cell Signaling, 5118s) or mouse derived LAMP1 monoclonal antibody (Cell Signaling, 15665), respectively. The bound antibodies were visualized using Alexa Fluor 555-labeled (red) and Alex Fluor 488-labeled (green) secondary antibodies. Cell nuclei were counterstained with 4′,6-diamidino-2-phenylindole (DAPI). Images were sequentially acquired on a confocal fluorescence microscope (Zeiss LSM 510 META; CarlZeiss, Thornwood, NJ).

### Cathepsin activity assay

The protease activity of cathepsins L, B and S in cells with or without GILT induction was determined by a fluorescence-based assay with selective substrate peptide conjugated with AFC according to the manufacturer’s protocol (Bio-Vision Mountain View, CA, USA) as described previously [18].

### Statistical analysis

All experiments were repeated at least three times with similar results achieved. For most figures, representative data or images were shown. Differences between control sample and tests were statistically analysed using one-way analysis of variance (ANOVA) with a Bonferroni adjustment for multiple comparisons. *p*-values less than 0.05 were considered statistically significant.

## Results

### GILT is expressed in lung epithelial cells and up-regulated by IFN-γ

**Figure 1.**
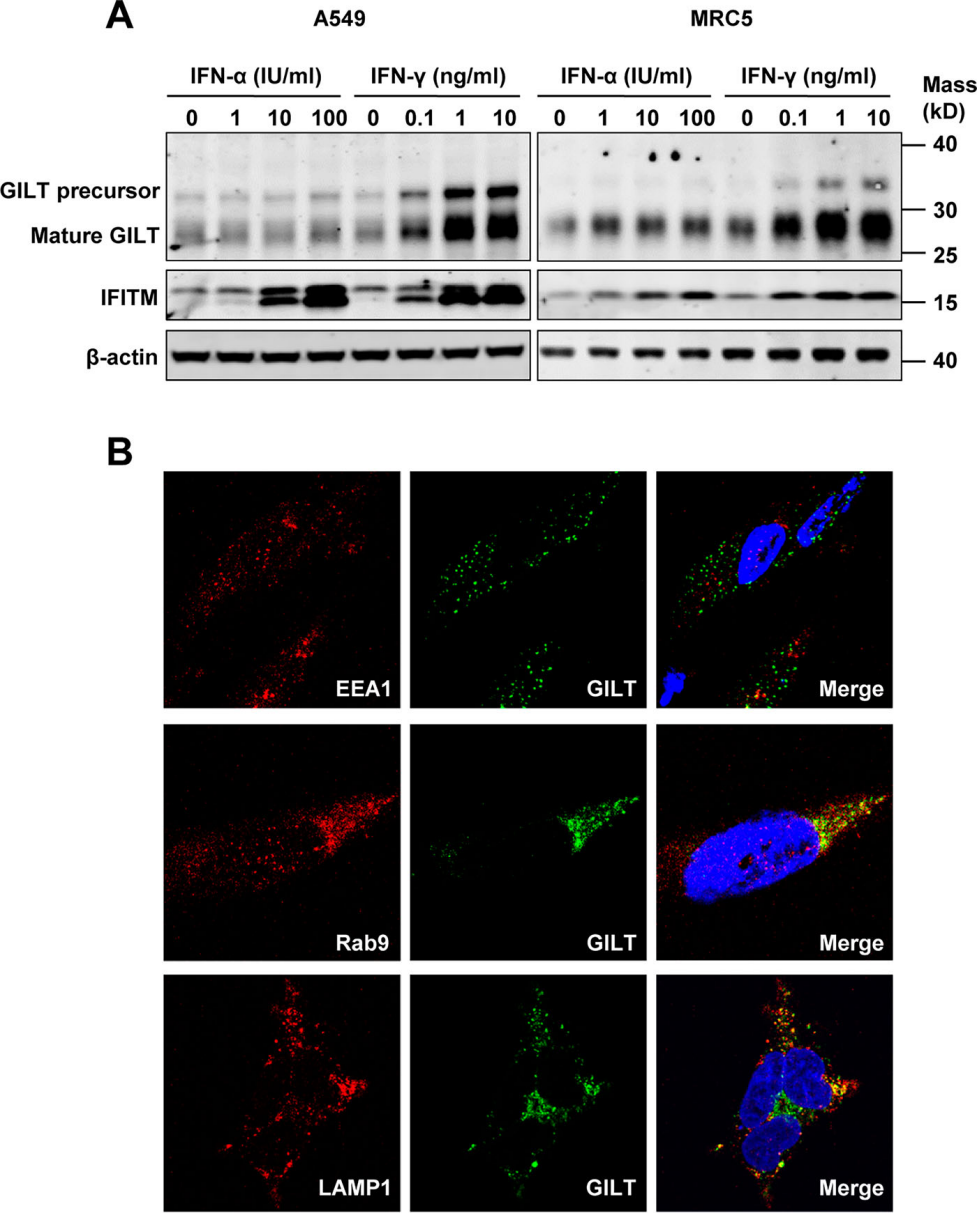
GILT expression is upregulated by IFN-γ in lung cancer cells and has a lysosomal distribution. A549 and MRC-5 cells were treated with the indicated concentrations of IFN-α or IFN-γ for 12 h. (A) The levels of intracellular GILT and IFITM proteins were determined by Western blot assay. β-actin served as a loading control. (B) The localization of GILT in MRC-5 cells treated with IFN-γ was detected by immunofluorescent staining with anti-GILT polyclonal antibody (green). EEA1, Rab9 or LAMP1 were visualized by immunofluorescent staining with respective antibodies (red). Cell nuclei were stained blue with DAPI.

GILT is synthesized as a precursor of 35-kDa soluble glycoprotein [15]. After sorting into endocytic compartments and lysosome, the N- and C-terminal propeptides are processed to produce a 30-kDa mature form protein [16]. As the only identified lysosomal thiol reductase that reduces the disulphide bonds of endocytosed antigens to facilitate their unfolding or degradation, GILT is constitutively expressed in multiple professional APCs [15,16]. However, the expression of GILT in other cell types, especially lung epithelia cells, is still unknown. To address this question, we assessed GILT expression in human lung cancer cell lines A549 and MRC-5 fibroblast cells. We found that both A549 and MRC-5 cells constitutively expressed GILT (Figure 1A). However, IFN-γ, but not IFN-α, dose-dependently induced GILT expression in both cell lines (Figure 1A). Consistent with that observed in professional APCs, microscopy studies revealed that GILT co-localizes with late endosome marker Rab9 and lysosome marker LAMP1, but not EEA1, an early endosome marker (Figure 1B).

### GILT inhibits viral entry mediated by envelope glycoproteins of SARS-CoV, Ebola virus and Lassa fever virus

**Figure 2.**
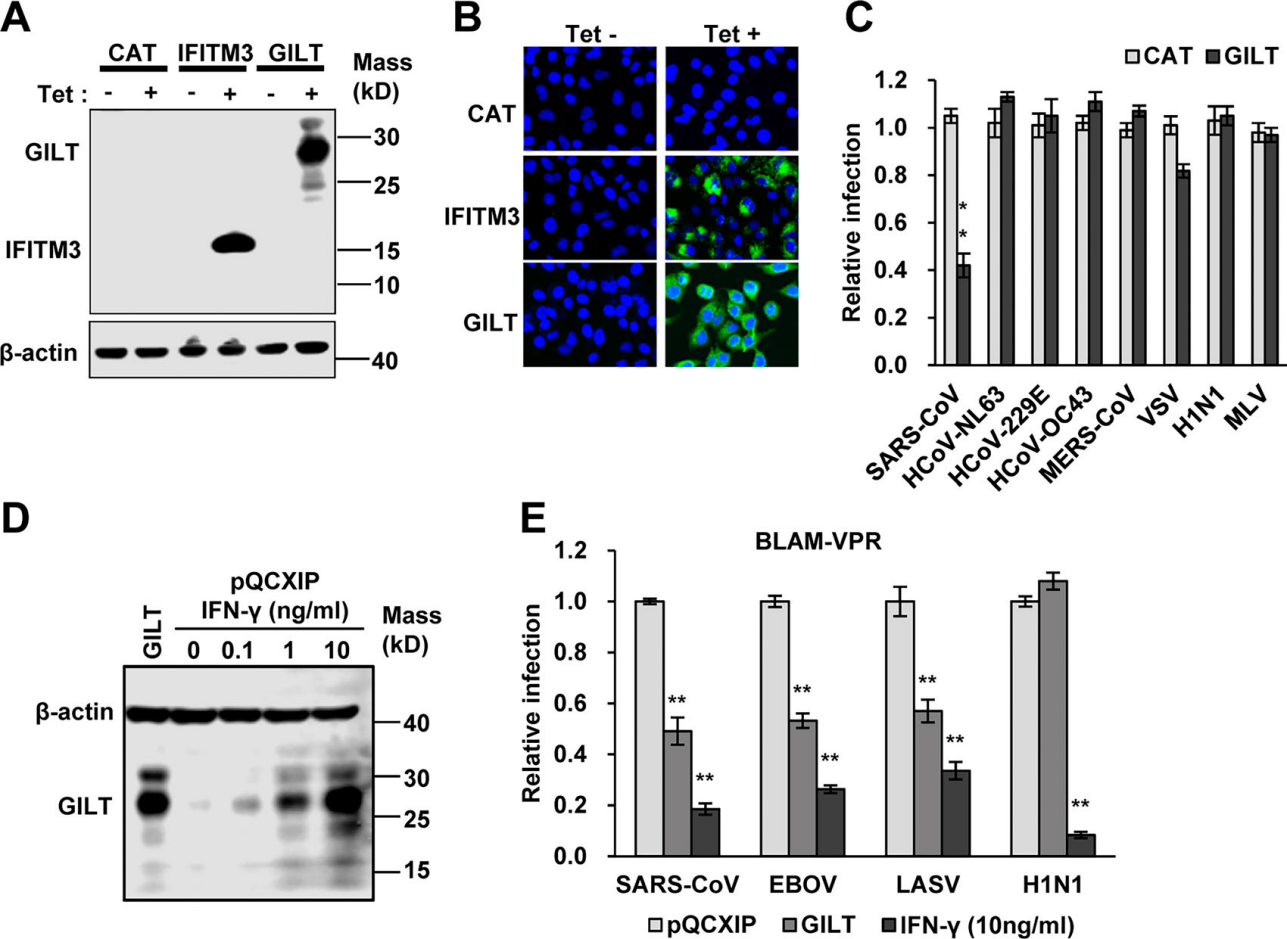
SARS-CoV, EBOV and LASV envelope glycoproteins-mediated infection is restricted by GILT. (A to C) FLIP-IN T Rex 293 cells that tetracycline (tet) inducibly express chloramphenicol acetyltransferase (CAT), GILT or FLAG-tagged IFITM3 proteins were cultured in the presence or absence of 1 μg/ml of tet for 24 h. The induced expression of GILT was detected by Western bot assay (A) and visualized by immunofluorescent staining (B). (C) The above-mentioned cells were then infected with indicated pseudoviruses. Pseudovirus infection was measured by luciferase assay at 48 h post infection (hpi). Relative infection represents the ratio of luciferase activity in cells treated with tetracycline over that in cells cultured without tetracycline. GILT significantly (**p < 0.001) inhibits the infection of SARSpp, but do not affect the infection of all other tested pseudoviruses. (D) The intracellular expression of GILT in A549 cell lines stably transduced with GILT or empty vector (pQCXIP) treated with IFN-γ at indicated concentration for 12 h was determined by a Western blot assay. (E) The entry mediated by GP proteins from SARS-CoV, EBOV, LASV and H1N1 in IFN-γ-treated A549 cells was determined by using the BlaM-Vpr assay. Cells were analysed by flow cytometry 24 h after infection and infectivity was determined by measuring cleaved CCF2 (Blue) from uncleaved CCF2 (Green). Relative infection efficiency was calculated by setting the infection of empty vector (pQCXIP) to 1.0. GILT significantly (**p < 0.001) inhibits the infection of SARSpp, EBOVpp and LASVpp, but do not affect the infection of IAVpp. Data are shown as the mean ± SD (standard deviation) of three independent experiments. *p < 0.05; **p < 0.001.

GILT facilitates the unfolding of internalized proteins containing disulphide bonds in lysosomes [37,38], the reduction of disulphide bonds in envelope glycoproteins of incoming virions by GILT may result in GP degradation or interrupt the GP-mediated membrane fusion and consequentially inhibit virus entry. To test this hypothesis, we established FLP-IN T Rex 293 cell line that expresses human GILT in a tetracycline (Tet)-inducible manner and determined its effects on the entry mediated by envelope GP from a panel of enveloped viruses, including five human coronaviruses (HCoVs), vesicular stomatitis virus (VSV), influenza A virus (IAV) H1N1 and murine leukaemia viruses (MLV). Tet treatment efficiently induced GILT expression, as judged by Western blot (Figure 2A) and immunofluorescence staining assays (Figure 2B). Interestingly, only the infection by lentiviral particles pseudotyped with spike protein of SARS-CoV (SARSpp) was significantly inhibited by GILT expression (Figure 2C). Considering that GILT is mainly localized in lysosomes and the SARS-CoV enters into the cytoplasm of host cells *via* lysosomes [26], we speculated that GILT may not restrain the viral entry that occurs at plasma membrane or early/late endosomes, but only impede the virus entry in lysosome. We thus further examined whether GILT could inhibit the entry of EBOV and LASV that enter cells in lysosomes [29,39,40]. To this end, A459 cells stably expressing ACE2, the receptor of SARS-CoV, were transduced with retroviruses expressing GILT or empty retrovector pQCXIP as a control, respectively. The expression of GILT in transduced A549 cells was at a level comparable to that induced by IFN-γ treatment (Figure 2D). As shown in Figure 2(E), both IFN-γ treatment and expression of GILT in A549 cells significantly inhibited the infection by Blam-Vpr lentiviral particles pseudotyped with envelope glycoprotein from EBOV, LASV and SARS-CoV. However, while IFN-γ treatment significantly inhibited Blam-Vpr lentiviral particles pseudotyped with IAV hemagglutinin 1 (H1) and neuraminidase (N1), expression of GILT did not, suggesting that other ISG(s) mediate IFN-γ suppression of IAV entry.

### Depletion of GILT protein enhances EBOV and LASV GP-mediated entry

**Figure 3.**
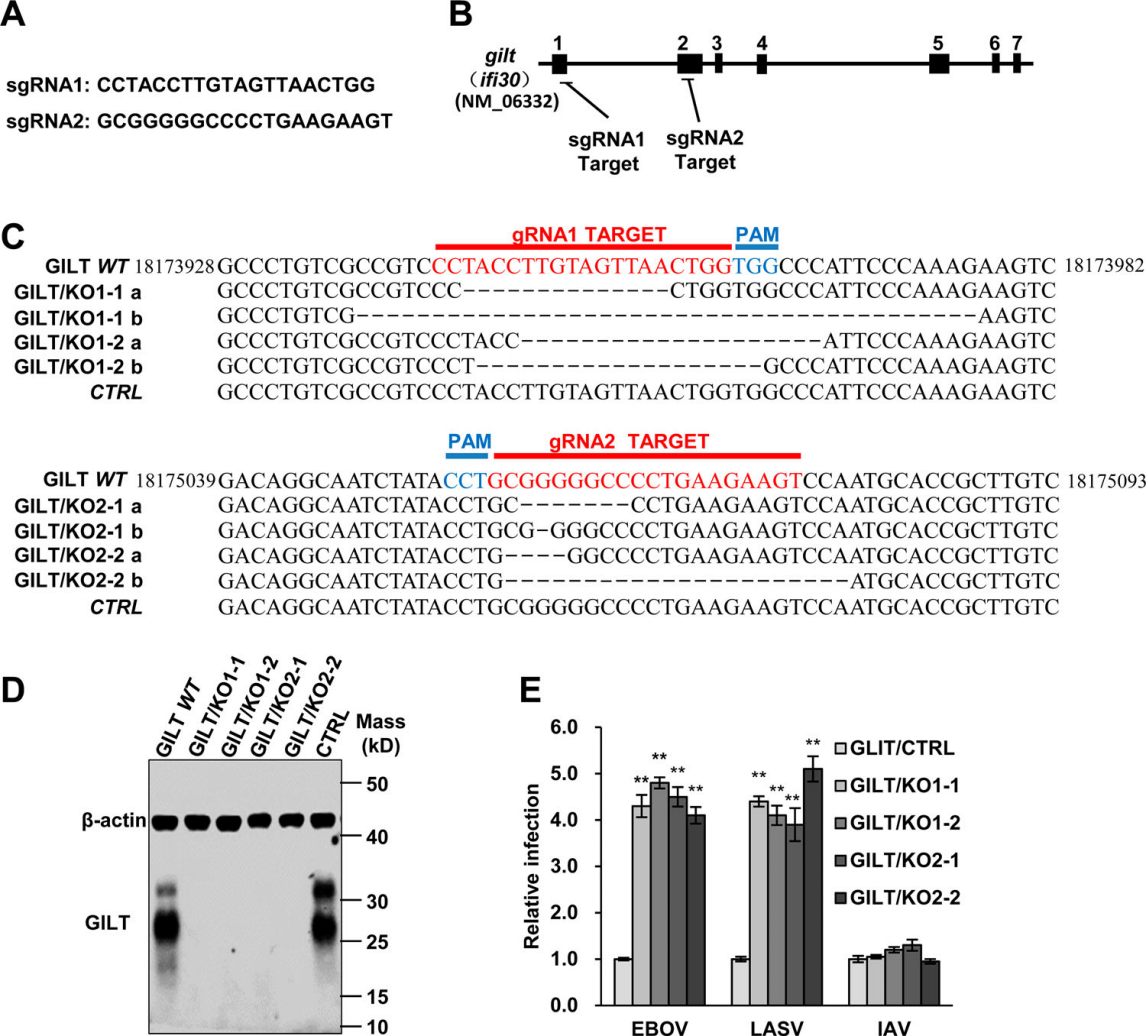
Knockout of GILT in THP-1 cells enhances viral entry of EBOV and LASV. (A) sgRNA sequences used in this study. (B) Schematic illustration of seven exons of *GILT* gene and guide RNA target regions. (C) Genomic DNAs from wild-type parental THP-1 (GILT *WT*) cells, GILT/KO THP-1 (GILT/KO1-1, 1–2 and GILT/KO2-1, 2–2) cells and empty vector control (*CTRL*) were isolated. gRNA-targeted regions in THP-1 chromosome were sequenced and aligned with GILT RefSeq. (NM_006332). (D) Cell lysates from indicated PMA-stimulated THP-1 clones were probed with a monoclonal anti-GILT antibody. β-actin severed as a loading control. (E) The above-mentioned THP-1 cells were infected with EBOVpp, LASVpp, or IAVpp, respectively. Pseudovirus infection was measured by luciferase assay at 48 hpi. Relative infection represents the luciferase activity normalized to that of THP-1 CTRL cells. Differences in relative pseudovirus infection between GILT/KO cells and THP-1 CTRL cells were statistically analysed (**p < 0.001). Data are shown as the mean ± SD of three independent experiments. *p < 0.05; **p < 0.001.

Previous studies have demonstrated that macrophages, the primary target cells of EBOV and LASV, constitutively express GILT [41]. To further validate the inhibitory effect of GILT on the entry of EBOV and LASV, we knockout the GILT in the myeloid cell line THP-1 to test whether depletion of GILT can augment the infection of EBOVpp and LASVpp. We generated THP-1 *gilt*-knockout cell clones, designated as GILT/KO cells, in which *ifi30* was disrupted by using three independent guide RNAs (Figure 3A and B). The genome sequencing analyses revealed that the gene of *ifi30* was ablated in the different clones of GILT/KO THP-1 cells (Figure 3C). Moreover, a high level of GILT expression was observed in PMA-differentiated THP-1 cells, while the GILT expression is abolished in GILT/KO THP-1 cells (Figure 3D). As anticipated, the GLIT knockout enhanced EBOVpp and LASVpp infection by approximately fourfold (Figure 3E). These results suggest that GILT is indeed a cell-intrinsic restriction factor to EBOV and LASV.

### Lysosomal sorting is essential for GILT activity of inhibiting viral entry

**Figure 4.**
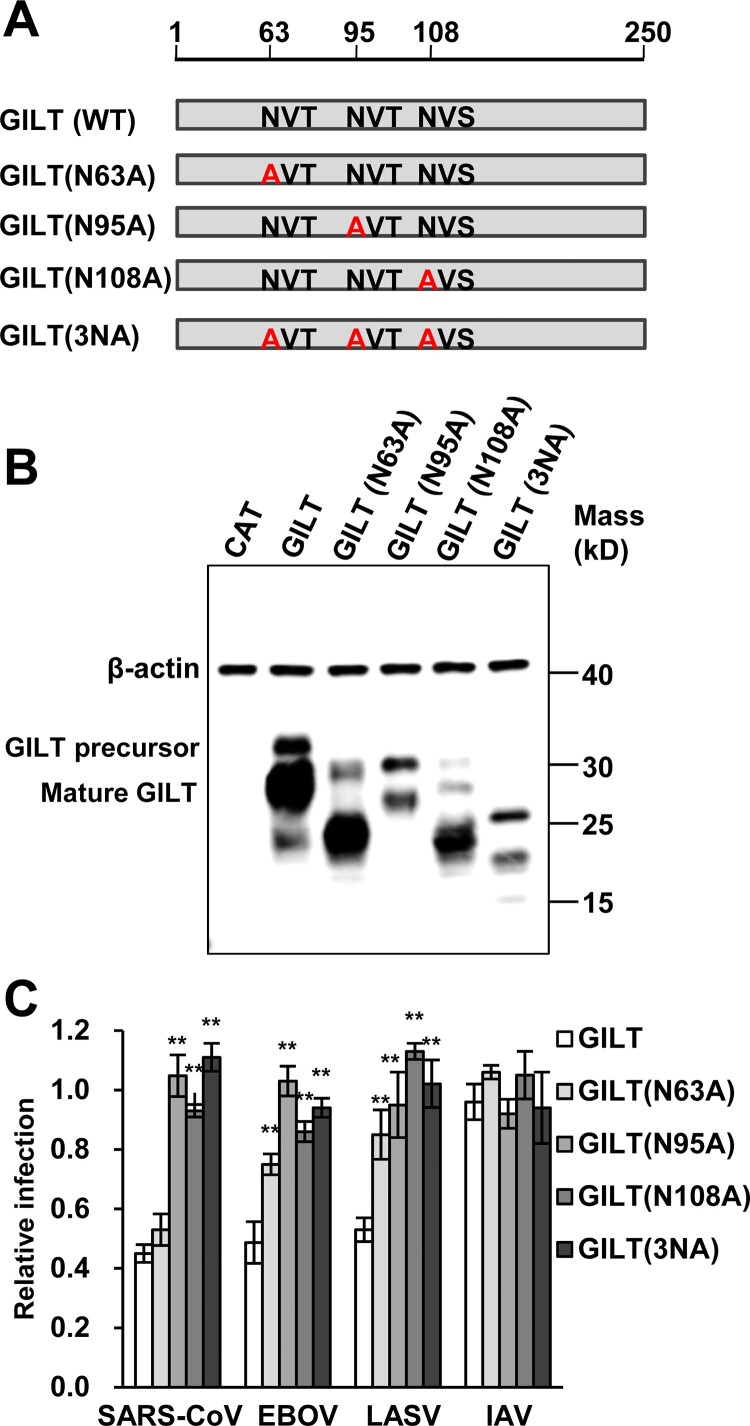
Mutations of N-linked glycosylation sites compromise GILT activity of inhibiting viral entry mediated by entry proteins from EBOV and LASV. (A) Illustration of GILT with three N-linked glycosylation sites and its mutants. (B) FLIP-IN T Rex 293 cells expressing GILT or indicated mutant GILT proteins were cultured in the presence of 1 μg/ml of tet for 24 h. The induced expression of indicated GILT mutants was detected by Western bot assay using anti-GILT polyclonal antibody, which recognizes both precursor and mature form. β-actin served as a loading control. (**C**) The above-described cell lines were infected with SARSpp, EBOVpp, LASVpp or IAVpp. Luciferase activities were determined at 48 hpi. Relative infection efficiency represents the luciferase activity in cells cultured with tetracycline normalized to that in cells cultured without tetracycline. Data are shown as the mean ± SD of three independent experiments. *p < 0.05, **p < 0.001 compared to wild-type GILT.

**Figure 5.**
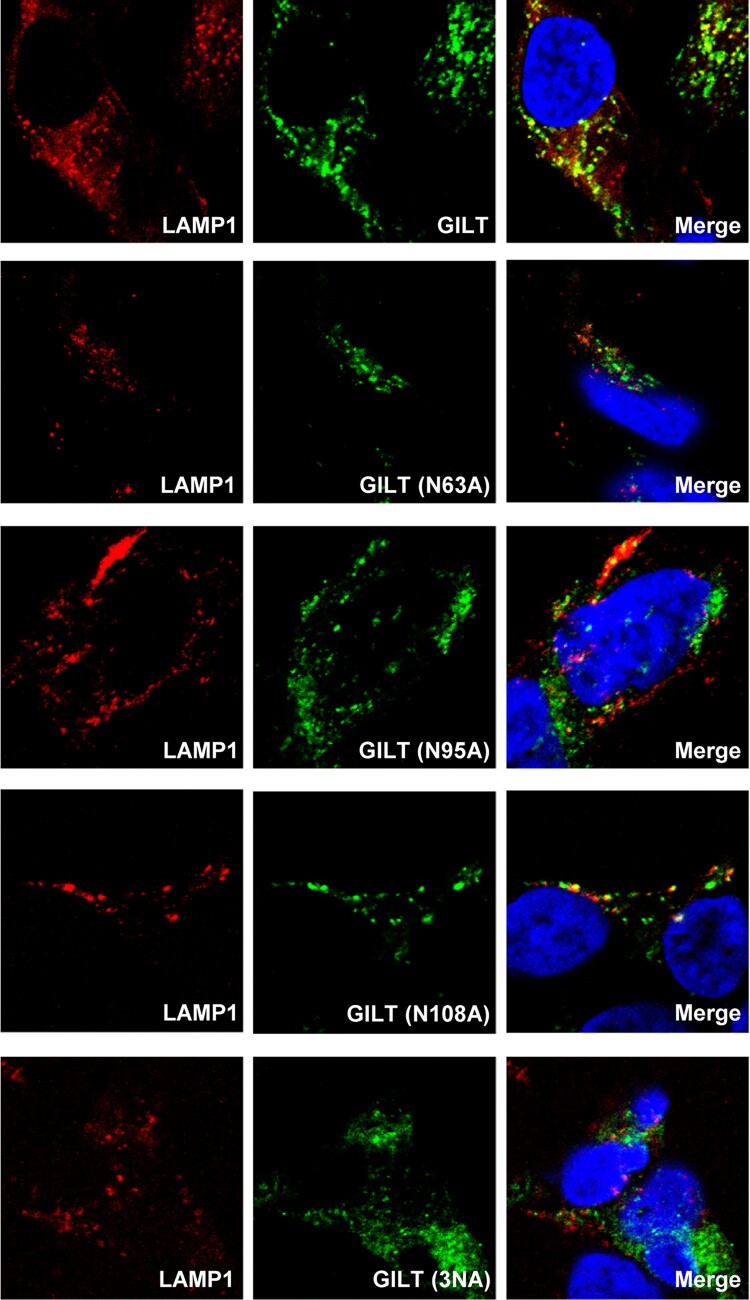
Mutations of N-linked glycosylation reduce GILT lysosomal localization. FLP-IN T Rex cells expressing the indicated wild-type and N-linked glycosylation mutant GILT proteins were treated with Tet for 24 h to induce GILT expression. The localization of wild-type GILT and its N-linked glycosylation mutants was detected by immunofluorescent staining with anti-GILT polyclonal antibody (green). LAMP1 was visualized by immunofluorescent staining with anti-LAMP1 monoclonal antibody (red). Cell nuclei were stained blue with DAPI (blue). More than 10 fields were examined, and the representative images of one or a few cells are presented.

Previous studies demonstrated that GILT is mannose-6 phosphorylated and sorting to lysosome via the mannose 6-phosphate receptor (M6PR) pathway [16]. To investigate whether N-glycosylation is required for GILT lysosome localization and antiviral function, three asparagine residues (N63, N95 and N108) of GILT were substituted by alanine individually or in combination (Figure 4A). Our results showed that the single alanine substitution of the three asparagine residues and their combined mutation (N3A) increased GILT migration by electrophoresis due to its impaired glycosylation and reduced the steady-state levels of GILT protein (Figure 4B). Compared to wild-type GILT, the mutant GILT protein exhibited impaired or lost activity to restrict the entry of SARS-CoVpp, EBOVpp and LASVpp (Figure 4C). Consistent with previous reports, wild-type GILT was observed in the perinuclear compartment and mainly co-localized with LAMP1. As anticipated, single mutation of glycosylation sites (N63A, N95A and N108A) partially reduced the co-localization of GILT with LAMP1, and triple mutation of glycosylation sites on GILT (3NA) completely abolished its co-localization with LAMP1 (Figure 5). These results collectively indicate that N-glycosylation of GILT is crucial for its lysosomal localization, which is essential for GILT to impede the viral entry occurring in lysosomes.

### Mutations of CXXC reductase motif abolished GILT-mediated restriction of viral entry

**Figure 6.**
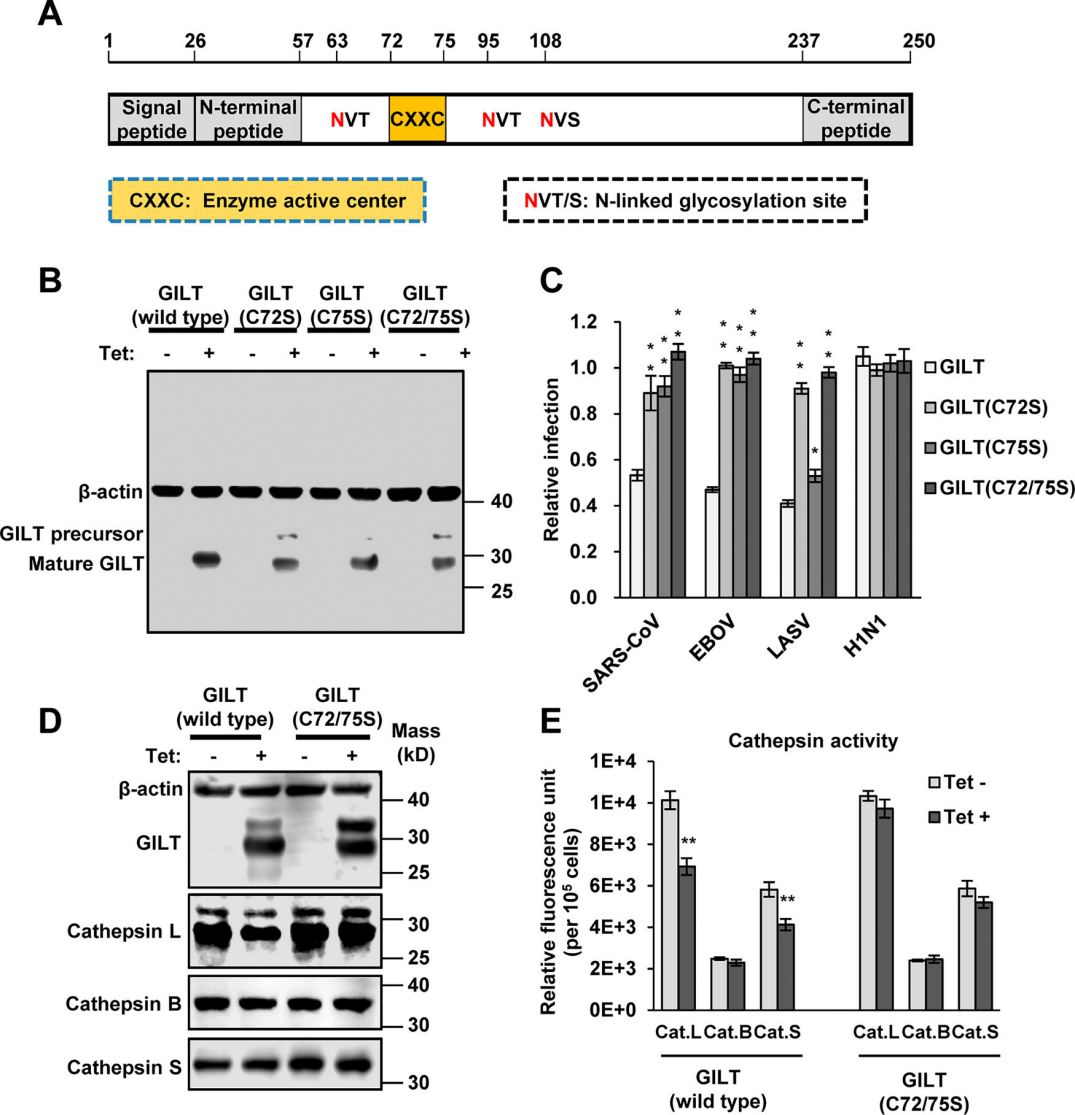
Cysteine mutations in thiol reductase active site compromise GILT activity to inhibit viral entry of EBOV and LASV. (A) Schematic representation of the GILT protein structural domains, motif of enzyme active centre, and N-linked glycosylation sites. (B) FLIP-IN T Rex 293 cells expressing GILT or indicated mutant GILT proteins were cultured in the presence or absence of 1 μg/ml of tet for 24 h. The expression of indicated GILT mutants was detected by Western bot assay using anti-GILT polyclonal antibody. β-actin served as a loading control. (C) The above-mentioned FLIP-IN T Rex 293 cells were infected with SARSpp, LASVpp, EBOVpp or IAVpp. Luciferase activities were examined at 48 hpi. Relative infection efficiency is the ratio of luciferase activity in cells cultured with tetracycline over that in cells cultured without tetracycline. Results are the means ± SD of three independent experiments. **p < 0.001 compared to wild-type GILT. (D) FLIP-IN T Rex 293 cells expressing wild type or mutant GILT proteins were left untreated or treated with tet for 24 h to induce the GILT expression. The expression of cathepsins L, B and S in FLIP-IN T Rex 293 cells were determined by western blot assay. (E) The cathepsin proteolytic activity in cells used in (D) were measured and presented as relative fluorescence unit. Results are the means ± SD of three independent experiments. **p < 0.001.

As a lysosomal thiol reductase, GILT has a conserved ^72^CXXC^75^ motif, the active site of thiol reductase, which is essential for GILT biological function (Figure 6A) [16]. To investigate the role of thiol reductase activity in GILT restriction of viral entry, we generated three mutant GILT with two residues of cysteine substituted with serine individually (C72S and C75S) and in combination (C72/75S). The results showed that all these three cysteine mutants were expressed to a level similar to that of wild-type GILT (Figure 6B). However, all mutant GILT lost activity to inhibit the infection of EBOVpp and SARSpp (Figure 6C). Compared with wild-type GILT, C75S GILT showed a reduced activity to inhibit LASVpp infection, but GILT with C72S and C72/75S mutations lost ability to inhibit LASVpp infection. Taken together, these results indicate that thiol reductase activity is essential for GILT to restrict the entry of EBOV, SARS-CoV and LASV.

More importantly, the expression of cathepsin L, but not cathepsin B and S, was reduced upon induction of wild-typed GILT, but not GILT with C72/75S mutations (Figure 6D). Because induction of GILT expression did not alter the levels of cathepsin L mRNA (data no shown), GILT might destabilize cathepsin L in a thiol reductase-dependent manner. In addition, GILT also reduced proteolytic activity of cathepsins L and S (Figure 6E). Since cathepsins L is an essential factor for the infectious entry of SARS-CoV and EBOV [42–44], our results thus imply that GILT inhibition of the cellular entry of SARS-CoV and EBOV is most likely by modulating the stability and enzymatic activity of lysosomal proteases, such as cathepsin L.

### Trypsin treatment circumvents GILT restriction of SARS-CoV S protein-mediated entry

**Figure 7.**
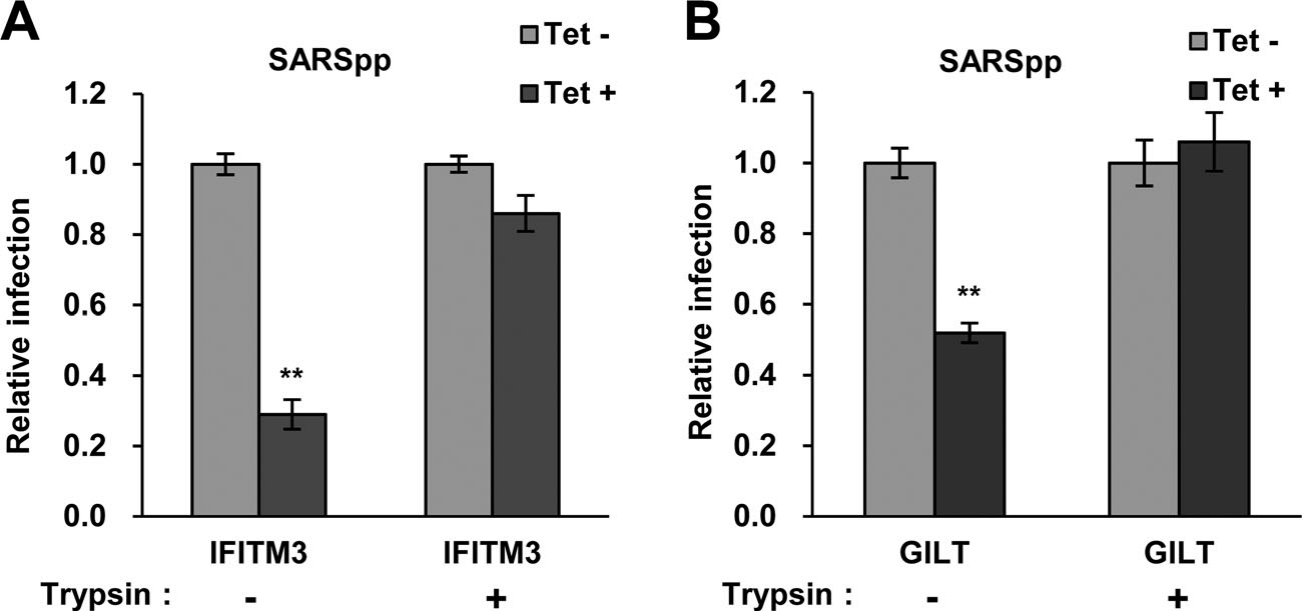
Trypsin treatment bypasses GILT restriction of entry mediated by SARS-CoV S protein. FLIP-IN T Rex 293 cells inducibly expressing IFITM3 (A) or GILT (B) proteins were left untreated or treated with tet for 24 h to induce the IFITM3 or GILT expression. Cells were then incubated with SARSpp at 4°C for 30 min and subsequently treated with TPCK-treated trypsin (5 mg/ml) or DMEM at 37°C for 15 min. The infected cells were maintained in complete DMEM and pseudovirus infection was determined by luciferase assay at 48 hpi. Relative infection represents the ratio of luciferase activity in cells treated with tetracycline over that in cells untreated with tetracycline. Results are the means ± SD of three independent experiments. Both IFITM and both GILT proteins significantly (**p < 0.001) inhibited infection by SARSpp.

SASR-CoV utilizes cathepsin L to activate S protein-mediated membrane fusion at lysosome [43]. Previous studies demonstrated that IFITM3-mediated restriction of SARS-CoV entry in lysosome can be bypassed by initiating membrane fusion at or near the plasma membrane *via* trypsin treatment of ACE2-bound pseudovirions [45]. To further confirm the location of GILT-mediated restriction, we examined the effect of trypsin treatment on SARS-CoV entry into GILT-expressing cells. In line with the results collected from IFITM3-expressing cells, the inhibitory effect on SARS-CoVpp infection by GILT was abrogated by trypsin treatment (Figure 7A and B). These results suggest that GILT cannot restrict SARS-CoV entry once it occurs at or near the plasma membrane, but only exerts restriction on viral entry in lysosomes.

## Discussion

As key antiviral cytokines, IFNs inhibit viral replication by inducing the expression of hundreds of ISGs that disrupt distinct steps of viral replication. Enveloped viruses enter cells by fusion with the plasma membrane or the membrane of endocytic vesicles and lysosomes. Thus far, several ISGs had been identified to modulate the entry of different viruses by altering the endocytic trafficking or membrane fusion. Our studies reported herein showed that GILT is a new ISG that restricts the entry of viruses invading the cytoplasm *via* lysosomes.

**Figure 8.**
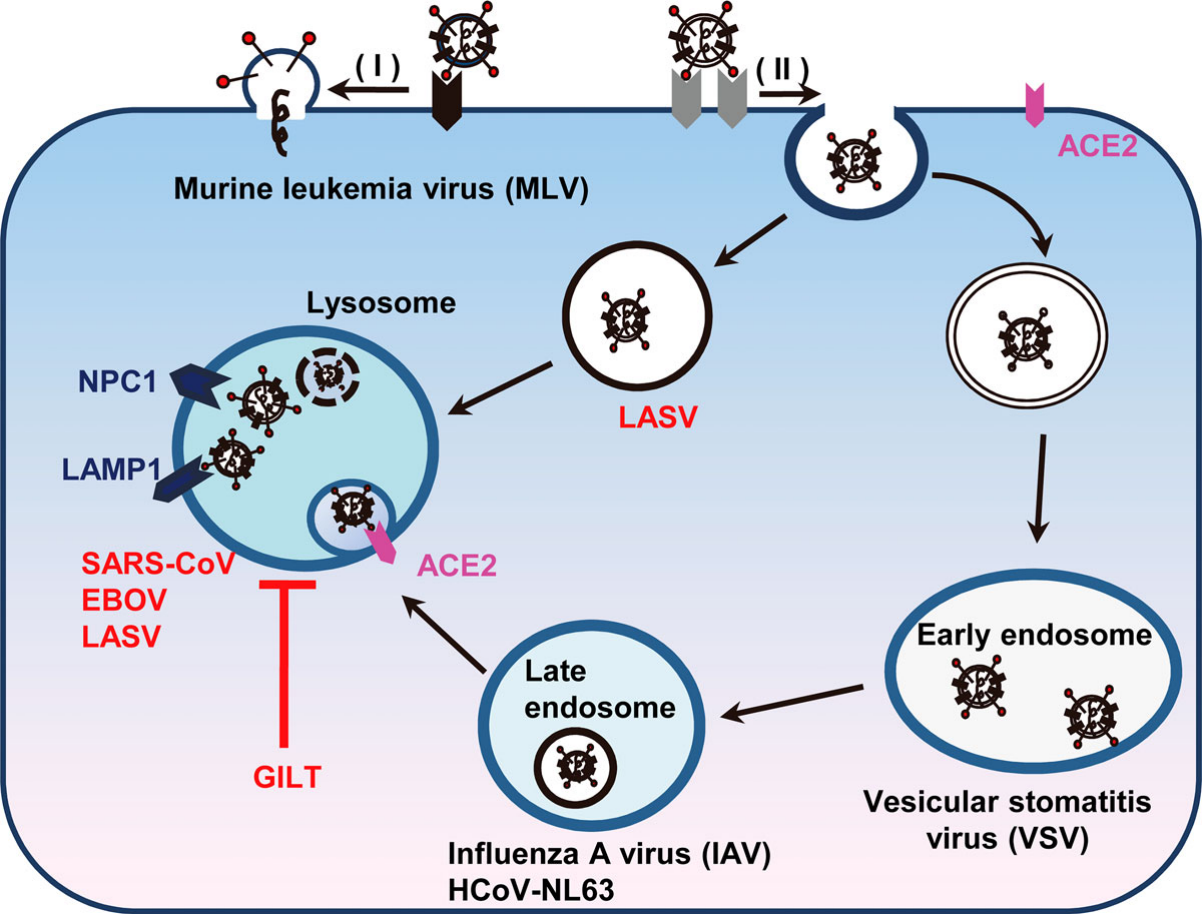
Schematic model for GILT-mediated restriction of viral entry in lysosome. Enveloped viruses enter cell at plasma membrane or escape from endocytic compartments via virus-host cell membrane fusion. While MLV that fuses at plasma membrane and vesicular stomatitis virus (VSV) and influenza A virus (IAV) that fuse at early endosome or late endosome are resistant to GILT-mediated inhibition, viruses including Ebola virus, Lassa virus and SARS-CoV, which fuse at lysosome, are restricted by GILT. NPC1: Niemann-Pick C1 protein (lysosomal receptor of Ebola virus); LAMP1: Lysosome-associated membrane glycoprotein 1 (lysosomal receptor of Lassa virus).

GILT, an IFN-inducible lysosomal thiol reductase, which reduces the disulphide bonds in internalized viral glycoproteins to facilitate their unfolding or processing and regulates the function of cathepsins in lysosomes, might affect the infection by certain viruses whose entry occur in lysosomes. Indeed, our studies revealed a unique profile of viruses that is susceptible to GILT-mediated restriction. GILT only inhibited the cellular entry of viruses including SARS-CoV, EBOV and LASV, which enter host cells via NPC1-positive lysosome (Figure 2). In contrast, other tested viruses such as MLV, VSV, IAV, HCoV-NL63, HCoV-229E and MERS-CoV, whose entry occur through cell surface, early endosome and late endosome, are refractory to GILT-mediated restriction (Figure 2). It is likely that GILT impedes viral entry in lysosome where the enzyme has the optimal activity of thiol reductase at low pH (pH = 4.5). In support of this scenario, our mutagenesis study demonstrated that the mutations altering the CXXC reductase motif or abrogating N-linked glycosylation, which is required for GILT lysosomal localization and optimal reductase activity, largely compromised GILT-mediated restriction of viral entry driven by GP proteins from SARS-CoV, EBOV and LASV (Figures 4 and 5). Of note, GILT might interfere cathepsin L metabolism and function to inhibit viral entry in lysosomes, which also relies on its thiol reductase (Figure 6). More importantly, trypsin treatment of SARS coronavirus activated the pseudovirions to directly fuse at the plasma membrane and circumvented GILT-mediated restriction (Figure 7). These data collectively indicated that GILT-mediated restriction of viral entry correlates with viral entry site and GILT optimal enzyme activity in the lysosome (Figure 8).

Diverse biological functions of GILT have been uncovered since this enzyme was identified. GILT contributes to T-cell development, modulates tolerance and autoimmunity, and improves cancer survival (reviewed in [27,38]). Importantly, GILT also has a critical role in microorganism infection. GILT plays a crucial role in antigen presentation by facilitating the unfolding of disulphide-rich viral glycoproteins to prime virus-specific T-cell activation in adaptive immunity [22]. Moreover, GILT functions as a host factor essential for *Listeria monocytogenes* infection by facilitating the phagolysosome escape through the reduction of bacterial hemolysins [46]. Furthermore, GILT inhibits dengue virus translation and replication by diminishing virus-induced autophagy [21]. In our study, we demonstrated that GILT is a critical restriction factor inhibiting viral entry of several pathogenic viruses. GILT is expressed in lung epithelial cells, a primary infection site of SARS-CoV. More importantly, a high level of GILT is expressed constitutively in macrophage, the primary target cells for filovirus replication. The knockout of GILT in THP-1 cells confers the cells higher susceptibility to EBOV and LASV GP-driven infection than parental THP-1 cells (Figure 3). These findings suggest that GILT may play an important role in restricting the infection by EBOV and LASV, although this finding should be validated with an authentic virus infection. Interestingly, it was reported previously that GILT restricted human immunodeficiency virus 1 (HIV-1) entry in a thiol reductase-dependent manner [47]. Because HIV-1 enters host cells at the plasma membrane or early endosome [48], it is very interesting to know how GILT restricts HIV-1 entry at those subcellular compartments. However, our preliminary studies indicated that expression of GILT did not inhibit the infection of GHOST cells, a cell line derived from human bone osteosarcoma, by HIV-1 strains utilizing either CCR5 or CXCR4 co-receptor (data not shown). Obviously, further studies are under way to investigate the discrepancy.

In summary, we demonstrated herein that GILT is an intrinsic restriction factor that arrests the lysosomal entry of several pathogenic enveloped viruses including SARS-CoV, EBOV and LASV, a function that is attributed to GILT’s optimal reductase activity in lysosomes. These findings extend our understanding of intrinsic immunity and provide a potential target for antiviral drug discovery.
